# *In vivo* soft tissue compressive properties of the human hand

**DOI:** 10.1371/journal.pone.0261008

**Published:** 2021-12-13

**Authors:** Victoria Spartacus, Maedeh Shojaeizadeh, Vincent Raffault, James Shoults, Ken Van Wieren, Carolyn J. Sparrey

**Affiliations:** 1 Mechatronic Systems Engineering, Simon Fraser University, Surrey, British Columbia, Canada; 2 International Collaboration on Repair Discoveries (ICORD), Vancouver, British Columbia, Canada; 3 Science Technical Center, Simon Fraser University, Burnaby, BC, Canada; Washington University in Saint Louis, UNITED STATES

## Abstract

**Background/Purpose:**

Falls onto outstretched hands are the second most common sports injury and one of the leading causes of upper extremity injury. Injury risk and severity depends on forces being transmitted through the palmar surface to the upper extremity. Although the magnitude and distribution of forces depend on the soft tissue response of the palm, the *in vivo* properties of palmar tissue have not been characterized. The purpose of this study was to characterize the large deformation palmar soft tissue properties.

**Methods:**

*In vivo* dynamic indentations were conducted on 15 young adults (21–29 years) to quantify the soft tissue characteristics of over the trapezium. The effects of loading rate, joint position, tissue thickness and sex on soft tissue responses were assessed.

**Results:**

Energy absorbed by the soft tissue and peak force were affected by loading rate and joint angle. Energy absorbed was 1.7–2.8 times higher and the peak force was 2–2.75 times higher at high rate loading than quasistatic rates. Males had greater energy absorbed than females but not at all wrist positions. Damping characteristics were the highest in the group with the thickest soft tissue while damping characteristics were the lowest in group with the thinnest soft tissues.

**Conclusion:**

Palmar tissue response changes with joint position, loading rate, sex, and tissue thickness. Accurately capturing these tissue responses is important for developing effective simulations of fall and injury biomechanics and assessing the effectiveness of injury prevention strategies.

## Introduction

Falls onto outstretched hands (FOOSH) are a leading cause of upper extremity injury. Falls during snowboarding, skiing, bicycle racing, in-line skating, ice skating, and certain gymnastics/acrobatics manoeuvers are common and can lead to traumatic injuries [1–6]. Upper extremities may be used to arrest a fall, which is useful for reducing injury to the head or torso [7, 8]; however, this results in the wrist being one of the most common injury sites after a fall [2, 9]. Fracture risk of the wrist depends on the magnitude and distribution of forces applied to the palmar surface and on the soft tissue response of the palm [10–13]. Increasingly, computational models [14–16] and crash test dummies [17–20] are being used to simulate falls to quantify impact mechanics and define injury risk; however, these models are limited by a lack of accurate soft tissue properties. Characterizing the *in vivo* response of the palmar soft tissue will help us better simulate fall mechanics, quantify injury risk and identify opportunities for injury prevention.

*In vivo* and *in vitro* tests have been used to characterize the mechanical behaviour of several human soft tissues [21–29]. Most soft tissues are known to display viscoelastic, anisotropic and non-linear responses [30–35]. Soft tissue properties are sensitive to several experimental variables including: strain rate [32, 36, 37], deformation [38], preconditioning [39–41], post mortem degradation [42–44], age [25, 45, 46], and sex [47, 48].

Studies characterizing *in vivo* soft tissues have primarily used quasi static loading or small deformations [38, 49], often due to equipment limitations. However, the nonlinear, viscoelastic nature of soft tissues means large deformation, high rate loading behaviour typical of falls and injuries cannot accurately be extrapolated from quasi-static or small deformation tests. Although falls onto hands are a common cause of injury and extensive research has focused on injury prevention, to the best of our knowledge, compressive behaviour for *in vivo* palmar tissue has not been characterized at loading rates or intensities representative of falls. In addition, including sex differences in tissue thickness and tissue mechanics are important for differentiating loading responses for females from males.

Recent work characterizing *in vivo* plantar soft tissues has highlighted the effect of joint position on soft tissue mechanics [25, 30]. Wrist positions are known to affect impact force during normal loading [50, 51] and Choi et al. [10] observed that peak force on the entire palm increased with increasing wrist angle during a forward fall. Furthermore, Penitente et al. [52] showed that during a gymnastic vault wrist angle affected peak impact force and loading rate. Extending the wrist, results in tension in the skin and soft tissues similar to the observations in the plantar tissues and as a result wrist positions are likely to affect palmar tissue mechanics. Therefore it is important to include the effect of wrist position when measuring palmar soft tissues.

The overall goal of this study was to characterize the *in vivo* compressive behaviour of palmar soft tissue under large deformation to quantify the soft tissue response experienced during a FOOSH. The specific objectives were to 1) characterize the response of *in vivo* palmar soft tissues to indentation testing; and 2) determine the effects of loading rate, joint angle, sex and tissue thickness on tissue mechanics. This study is the first to experimentally characterize the high rate, large deformation indentation response of *in vivo* palmar soft tissues. These properties provide important calibration and validation data for fall simulations and understanding and quantifying injury risk.

## Materials and methods

### Participants

Fifteen healthy right handed young adults (8 males, 7 females) participated in this study (mean age = 26.53 years (SD = 2.47), mean height = 1.7 m (SD = 0.1), mean weight = 67.17 kg (SD = 12.48) and mean BMI = 23.06 (SD = 2.54)). Participants with acute or chronic pain, active disease state that could affect soft tissue stiffness or thickness, or a history of hand/wrist surgery two years prior the experiment were excluded. All participants provided written informed consent. The experimental protocol was approved by the Department of Research Ethics at Simon Fraser University.

### Equipment

The mechanical characterization was completed using a precision linear actuator (Bose LM 3200). Although the system includes software limits and an emergency stop button, a physical stop plate was mounted under the Bose actuator to limit the movement to ensure participant safety.

A handheld linear array transducer (L7 (4–13 MHz), Clarius Mobile Health) was used to measure the soft tissue thickness of the palmar surface. For measurement of soft tissue the depth setting of 3 cm was kept constant.

### Protocol

Ultrasound measurements were acquired with the hand palmar side up on the examining table with the thumb in a relaxed position. The ultrasound probe was placed on the palmar surface at the base of the thumb, longitudinal to the trapeziometacarpal joint [53] and still frames were captured. Soft tissue thickness was measured over the trapezium where indentation tests were performed. This location was chosen because according to Choi et al. [10] the trapezium is located in the danger zone where highest force occurs during a fall. Two measures were taken and an average of the soft tissue thickness was used to determine the indentation test parameters.

**Fig 1:**
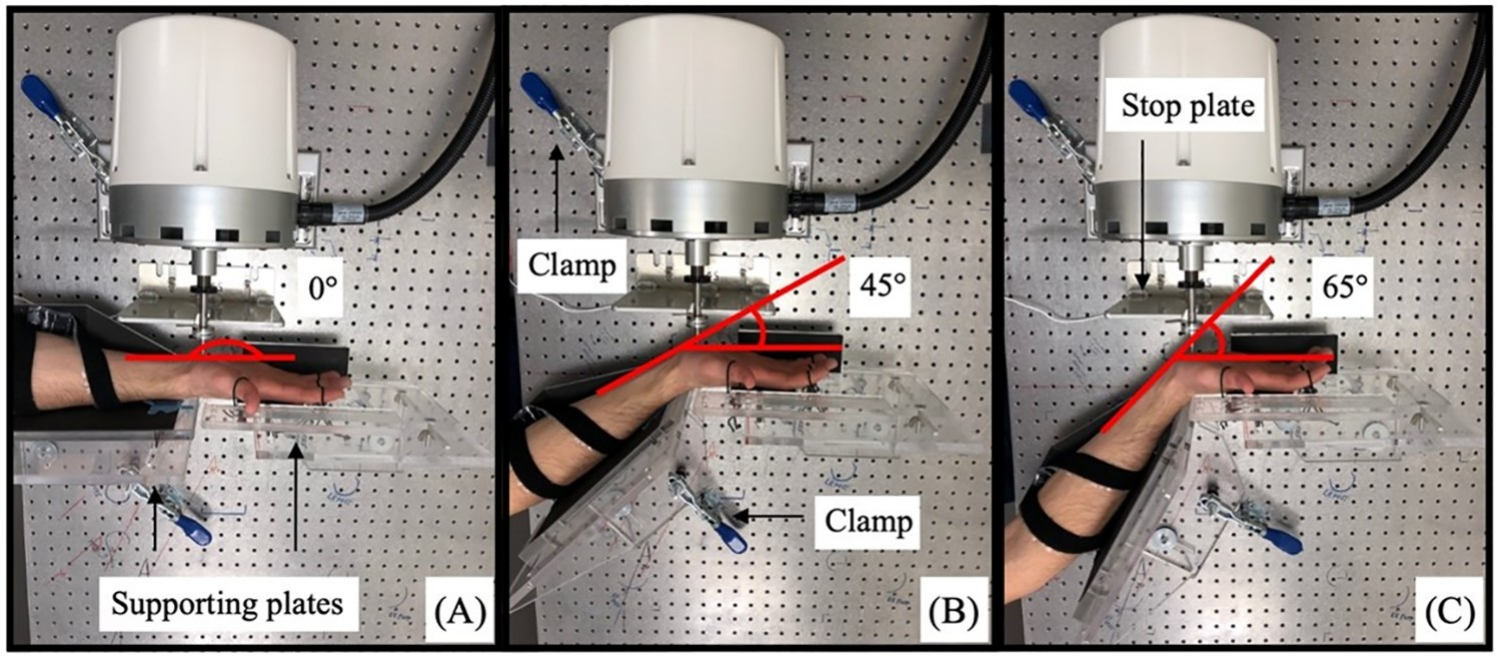
Indentation angles, (A) 0° extension for wrist in neutral position, (B) 45° extension and (C) 65° extension.

Indentation tests were performed on the dominant hand (right hand) at three different wrist angles: 45 degrees extension, 65 degrees extension and 0° degree extension for the wrist in neutral position (Fig 1) and two test frequencies (0.2 Hz and 20 Hz). To hold the hand in these positions during the experiment, two custom machined angled plates were used to provide stable support for the participants and to keep the palm perpendicular to the indentation probe.

**Fig 2:**
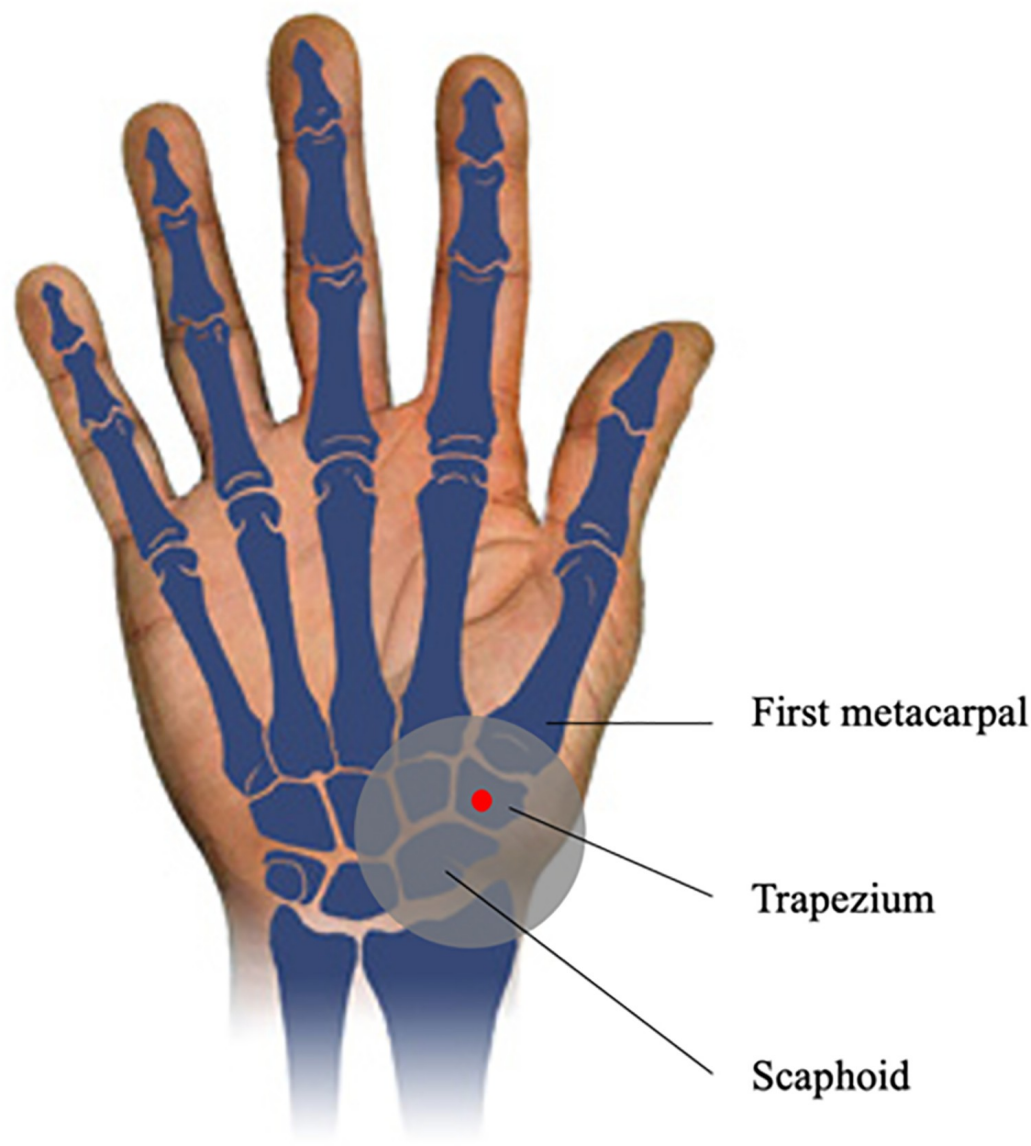
Palmar location tested during the indentation trials (red dot). The dark gray zone represents the danger zone where highest force occurs during a fall [10].

The participant was seated and able to remove and relax their hand and arm between test cycles. The palmar location tested (above the trapezium) was marked with a dot of non-permanent marker for repositioning between tests (Fig 2).

**Fig 3:**
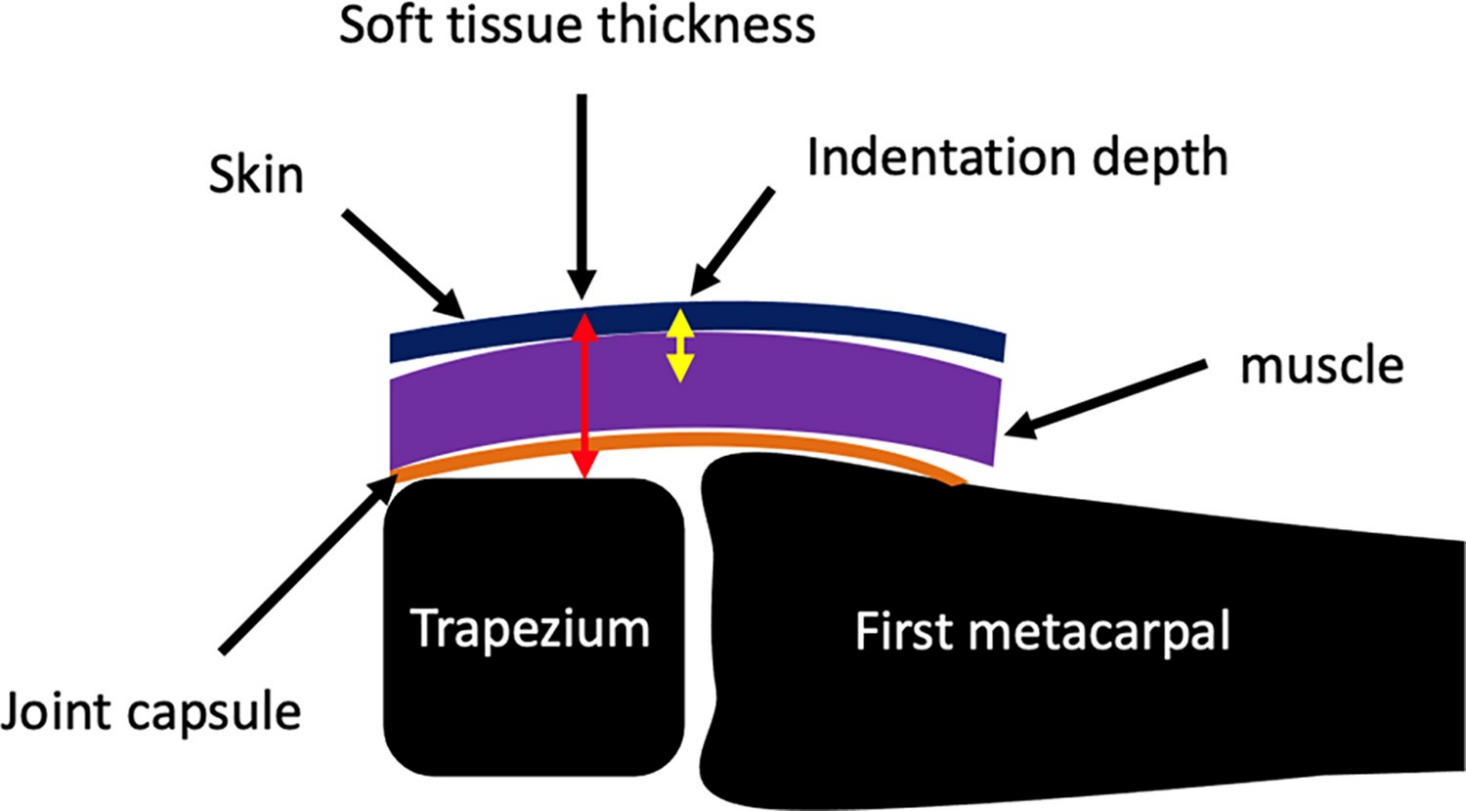
Schematic view of trapeziometacarpal joint anatomy.

For the indentation tests, a 3-mm diameter cylindrical flat-ended indenter tip was used [54, 55]. The tests were conducted at two different frequencies 0.2Hz and 20Hz to 50% of the participant’s soft tissue thickness in order to identify the viscoelastic properties of the tissue [30]. A touch force of 0.1N was used to establish a consistent starting position for each test. Twenty-two cycles were performed for each test. The fifth cycle to the twenty-first cycle were averaged to represent the stable tissue response. The first four cycles preconditioned the tissue and removed the effects of prior loading on the observed response. During each trial the order of wrist positions and indentation frequencies were randomized to avoid the effect of loading history. The trapezium is covered by different layers of soft tissue which include skin and muscle (Fig 3). The indentation depth of 50% of soft tissue thickness behaviour incorporates structural properties of the muscle and skin.

Force and displacement were recorded during the indentation trials. Stiffness at small (0–5%) and large (25–30%) deformation were calculated by finding the slope of the force-displacement curve at the respective intervals. Energy absorbed was calculated as the area under the force-displacement curve using trapezoidal numerical integration.

Statistical analyses were conducted to assess the effects of independent variables (loading rate, wrist angle, sex, and tissue thickness) on dependent variables (peak force, absorbed energy, and tissue stiffness). Analyses of variance were used to evaluate differences in peak force and energy absorbed between subjects. Non-parametric tests (Friedman) were used to test for differences in peak force and energy absorbed between wrist angles. Non-parametric paired test (Wilcoxon) tests were used to test for differences in peak force, energy absorbed and tissue stiffness between loading frequencies. Non-parametric ANOVA tests (Kruskal-Wallis) were used to evaluate differences in peak force and energy absorbed between male and female subjects. To differentiate tissue thickness effects from the effects of sex, we observed tissue behaviour according to soft tissue thickness. The participant cohort was divided in three groups (< 2.5 mm, 2.5–3.0 mm and > 3.0 mm) and the Spearman correlation test was used to test relationships between soft tissue thickness and each outcome variable. All analyses were conducted with the SPSS 25.0, using a significance level of α = 0.05.

## Results

**Fig 4:**
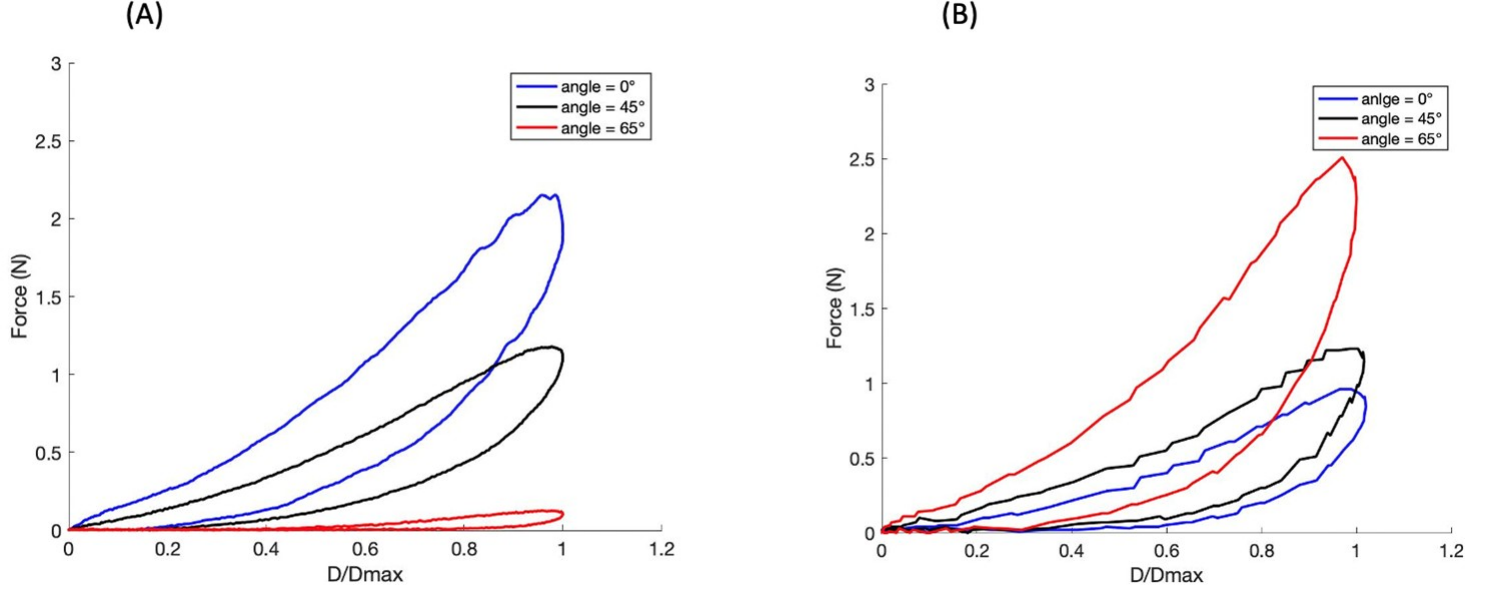
Representative force-displacement curves for one subject at low (A) and high (B) frequencies show marked hysteresis in the unloading responses.

The palmar tissue response was nonlinear and rate dependent (Fig 4). There was marked hysteresis in the unloading response at both high and low rates.

**Fig 5:**
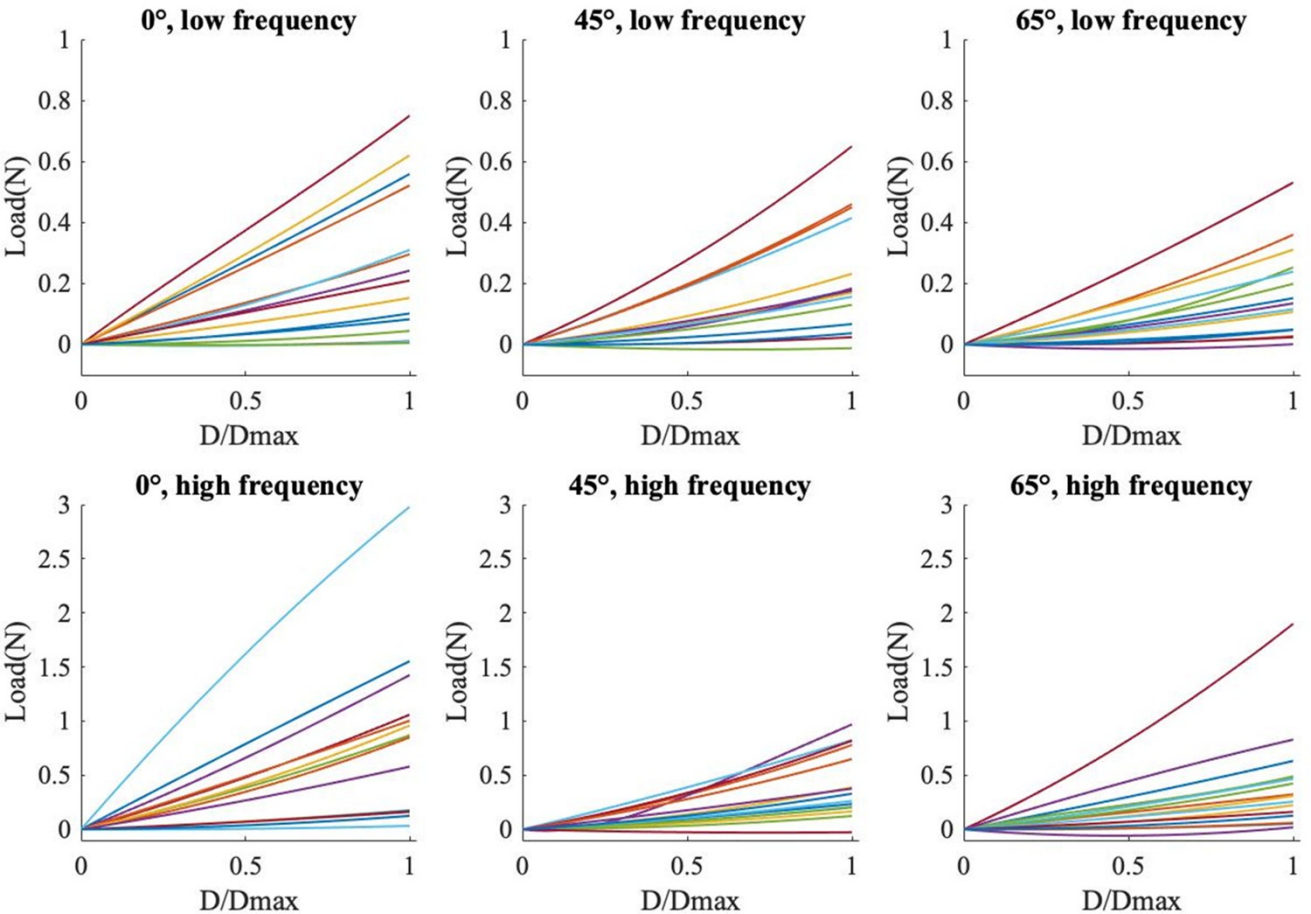
Force displacement curves for subjects and angles at low and high frequency normalized to peak displacement show high inter-subject variability.

At high frequency, the displacement of the probe did not reach 50% of tissue thickness due to limitations of the BOSE test system. Therefore in the comparative analysis of high and low loading rates compression to 31% (± 0.2) of soft tissue thickness was considered. There was high variability in peak force within each test group with coefficients of variation ranging from (39–110%) (Fig 5).

Palmar tissue response was displacement dependant (Table 1). Tissue stiffness at small deformation was significantly lower than stiffness at large deformation. At low frequency, stiffness at large deformation was more than 50% higher than at small deformation (p = 0.001). At high frequency, stiffness at large deformation was more than 40% higher than at small deformation (p<0.003). Looking at the extreme value ranges, individuals with the lowest or the highest stiffness are not always the same throughout the tests (i.e. the effects of position, and loading rate were not consistent across all individuals).

**Table 1:** Average of stiffness at small deformation (S.D.) and large deformation (L.D.) for males, females, and all participants.

		0.2Hz				20Hz			
		Stiffness S.D. (N/mm)	Range	Stiffness L.D. (N/mm)	Range	Stiffness S.D. (N/mm)	Range	Stiffness L.D. (N/mm)	Range
**Female**	**0°**	0.22	0.02–0.76	0.56	0.11–1.78	0.75	0.03–2.18	1.14	0.37–1.89
**Male**		0.30	0.004–0.62	0.52	0.15–0.94	0.58	0.01–1.51	1.13	0.19–2.45
**Total**		0.26	0.004–0.76	0.54	0.11–1.78	0.66	0.01–2.18	1.13	0.19–2.45
**Female**	**45°**	0.22	0.005–0.77	0.56	0.16–1.58	0.33	0.08–0.84	0.89	0.47–1.83
**Male**		0.23	0.009–0.51	0.61	0.15–1.16	0.34	0.04–0.77	1.56	0.24–7.15
**Total**		0.23	0.005–0.77	0.58	0.15–1.58	0.34	0.04–0.84	1.25	0.24–7.15
**Female**	**65°**	0.21	0.02–0.54	0.54	0.17–1.15	0.39	0.0007–1.6	1.43	0.32–3.81
**Male**		0.12	0.006–0.3	0.34	0.15–0.52	0.28	0.008–0.72	0.69	0.35–1.21
**Total**		0.16	0.006–0.54	0.43	0.15–1.15	0.32	0.008–1.6	1.04	0.32–3.81

**Fig 6:**
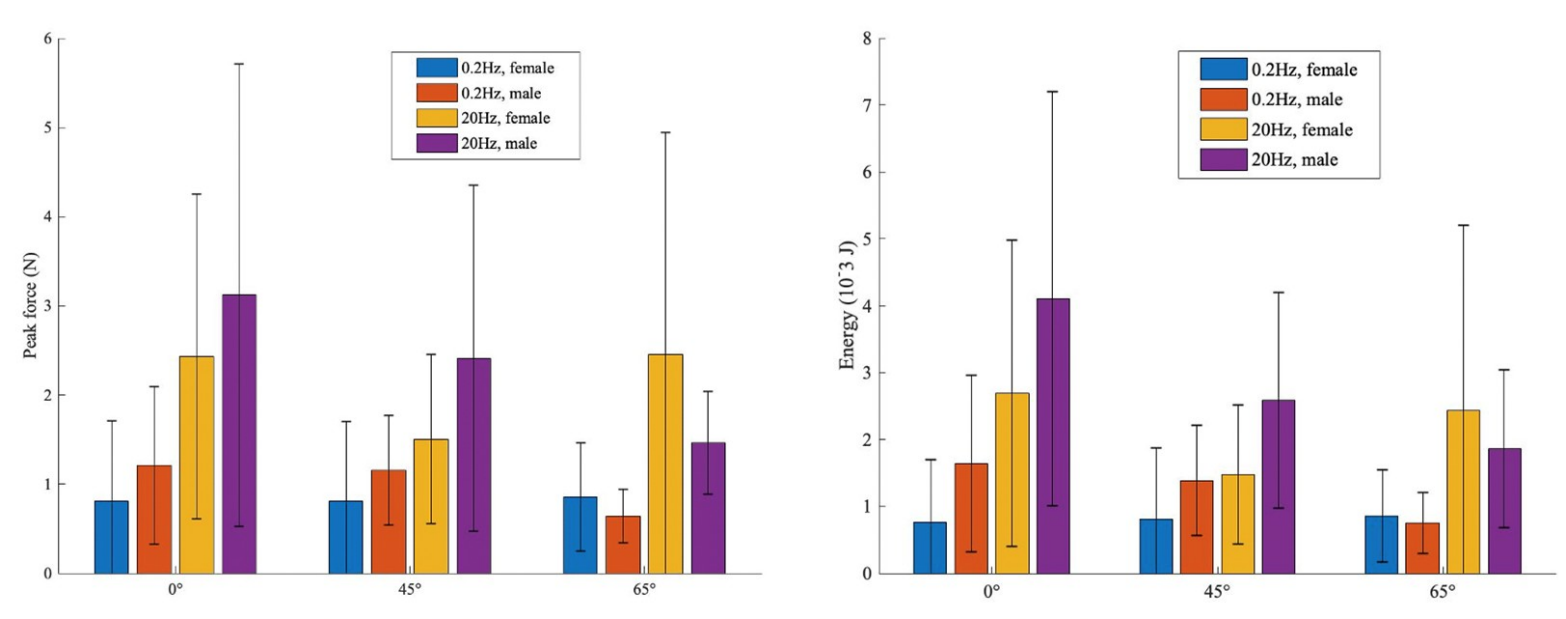
Average of peak force (N) and energy absorbed (10^-3^J) at 31% soft tissue compression for male and female for all positions and both frequencies.

The peak forces, measured at 31% displacement showed a significant effect of loading rate (p = 0.001). The peak force at maximum displacement was 2–2.75 times higher for high frequency than for low frequency for all joint positions (p = 0.001) (Fig 6). At both frequencies, the peak contact force decreased with increased wrist extension but the effect was not statistically significant. In addition, at both frequencies and at each position the average peak force was greater for males than females except for maximum extension but the differences were not significant due to the large variation with the groups (p<0.2) (Fig 6).

Similar to the peak force observations, the energy absorbed during contact was highly variable between individuals but showed that increased loading rate increased the energy absorbed for each position by 1.7–2.8 times (angle 0°: p = 0.001, angle 45°: p = 0.001 and angle 65°: p = 0.001) (Fig 6). There were trends of wrist position decreasing energy absorption with increased extension but no significant effect was found. There were also trends of effects of sex on energy absorbed but it was not significant. At both frequencies the average energy absorbed was higher for males than females except at the angle 65° (Fig 6).

**Fig 7:**
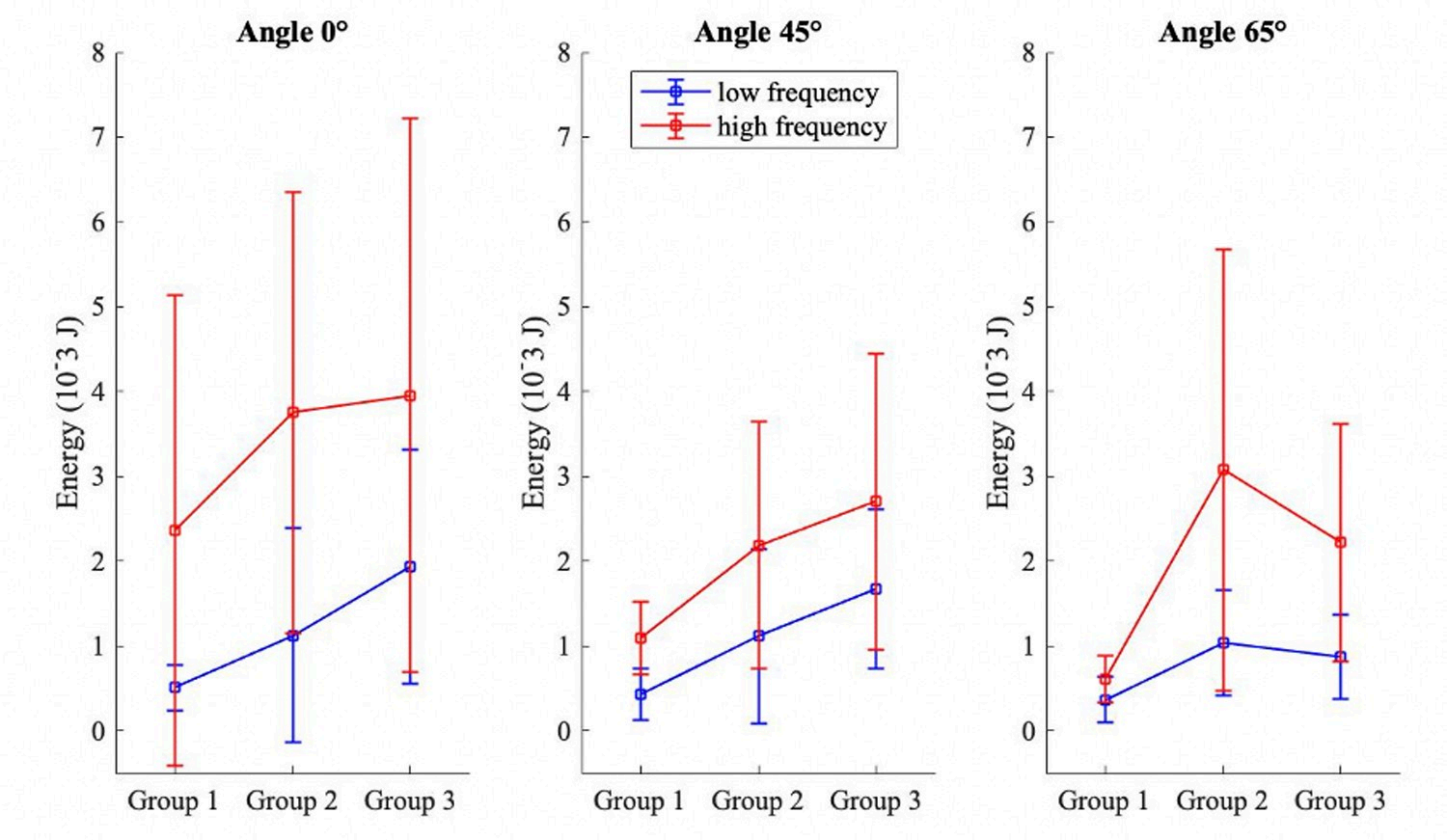
Average of peak force at different angles for high and low frequency grouped by tissue thickness (group 1 < 2.5 mm, 2.5 mm < group 2 <3.0 mm and group 3 > 3.0 mm).

Palmar tissue thickness was positively correlated with weight (R = 0.71, p<0.01), height (R = 0.62, p<0.05) and BMI (R = 0.54, p<0.05). Palmar tissue thickness was different between males and females; the average male tissue thickness was 20% greater than female (p<0.01). There was a trend of an effect of tissue thickness on peak force. At high frequency, peak force increased between group 1 and group 2 and decreased for group 3. Peak forces between tissue thickness were significantly different only at high frequency at 65° between group 1 and group 2 (p = 0.04). Peak force was 51% higher in group 2 than in group 1 (Fig 7). At low frequency and angle 65°, a similar trend was observed, peak force increased between group 1 and group 2 and decreased for group 3. However at 45° and the neutral position, peak force increased with the increased tissue thickness without significant differences (Fig 7).

**Fig 8:**
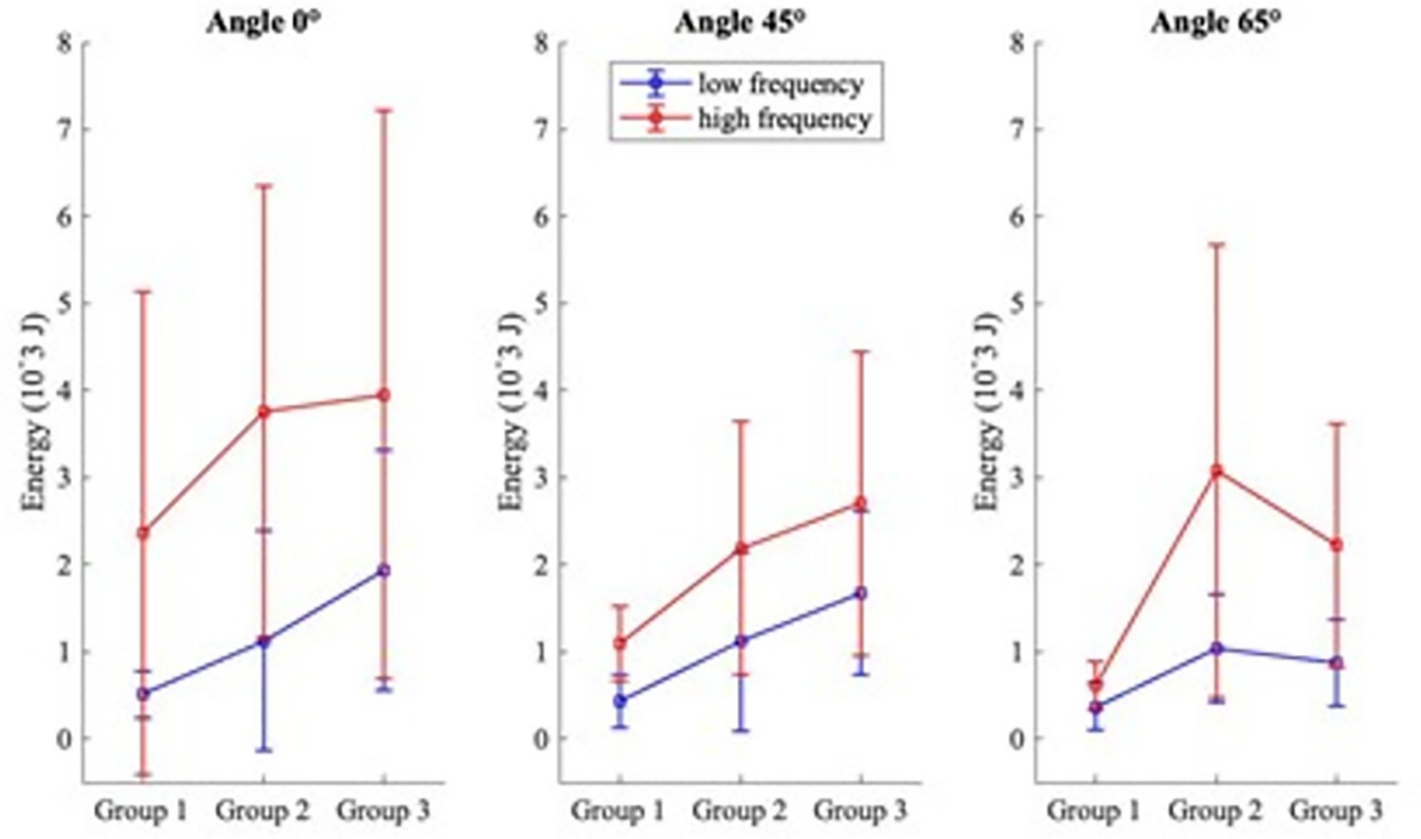
Average of energy absorbed on different angles regarding various tissue thicknesses.

Similar to the peak force observations at high frequency at 65°, the energy absorbed increased between group 1 and group 2 and decreased for group 3 (Fig 8). Energy absorbed showed a significant effect of tissue thickness, between group 1 and group 2 (p = 0.04). Energy absorbed was than 73% higher in group 2 than in group 1 at high frequency at 65°. At the neutral position and the angle 45°, energy absorbed increased with increasing tissue thickness at both frequencies without significant differences (Fig 8).

## Discussion

Falls onto outstretched hands are common and a leading cause of upper extremity injury. Computational models are increasingly being used to simulate fall events and determine injury risk. The mechanical loading during a fall onto the hands is, in part, affected by the tissue properties used to represent the palmar soft tissues. However, those tissue properties have not been measured for *in vivo* tissues. *In vivo* characterization is critical in this body location due to the amount of muscle covering the trapezium. Cadaveric tissues are often used in biomechanics; however post mortem changes in tissue properties governs the degree to which the cadaver response represents live human response. Relatively small variations have been found on post mortem bone [56, 57], ligament [58, 59], tendon [60], skin [61], or articular cartilage [62] from their live mechanical properties. In contrast, large changes have been found in post mortem skeletal muscle properties [63–66]. The changes observed in skeletal muscle properties may represent the largest limitations of the cadaver model in reproducing biofidelic mechanical responses. Correct identification of the *in vivo* properties of the palmar soft tissue is a necessary step in elucidating the biomechanical and functional morphology of the human hand in fall simulations and computational models. In this study, we conducted *in vivo* dynamic indentation to quantify the compressive characteristics of palmar soft tissues for the first time.

### The effect of wrist position on compressive mechanics

We found that wrist angle had limited effect on peak force or energy and that while there were trends in the behaviour the results were not statistically significant. When looking at the responses to wrist angle for each individual, the effect was inconsistent; for some participants increasing wrist extension decreased peak force and energy, while for others the opposite response was observed. At low frequency, peak force and energy absorption decreased slightly with increasing wrist angle. At high frequency, peak force decreased with the increasing of wrist angle. However the energy decreased between the neutral position and the angle 45° and increased for the angle 65°. Our results are opposite to Chen et al. [30] and Teoh et al. [25] who found that plantar second metatarsal head soft tissue stiffness increased with metatarsophalangeal joint dorsiflexion; however, these studies were completed at low loading rates (9.5 mm/s) and showed less inter-subject variability than we observed in our participants. In the Chen and Teoh studies the tissue was substantially preloaded (pressure was applied to the whole toe area) before focal indentation testing occurred whereas our study used a limited preload (0.1 N) to reflect the complete load response of the tissue. Theoretically, increasing wrist extension would increase the tension in skin tissue and an increase of peak force with wrist extension should be expected. However in our situation the indenter tip applied a load on the area above the trapezium where the intrinsic muscles of the thumb are located, more specifically the abductor pollicis brevis and the opponens [67, 68]. In the experimental setup the thumb and fingers were held in specific positions where muscles were stretched. Increasing wrist extension could increase the stretching of the muscles. Muscles stretched have not shown to have less stiffness than muscle contracted [69, 70]; however, during stretching a greater muscle length [69] and decreased muscle thickness occurs. Decreasing muscle thickness could allow the skin and underlying ligaments, joint capsule or fat to play a larger role in the compressive response that could explain the decrease in the peak force.

### Effect of loading rate

Peak force and energy were highly dependant on loading rate–showing up to a 3 fold difference between high rate (20 Hz) and low rate (0.2 Hz) loading. Dependence on loading rate was also found for peak force and energy for males and females separately. This result is in accordance with our general understanding of tissue viscoelasticity and the many studies that have characterized isolated tissue samples. It aligns with Negishi et al. [71] who measured *in vivo* heel pad biomechanical properties. They showed that the peak force increased with the increasing of indentation rate–though the magnitude of the increase was much lower than observed in this study due to the smaller range of loading rates explored. Loading rate dependence of soft tissue mechanical properties was also observed on plantar cadaver studies [72, 73]; however their results showed a smaller effect of loading rate (less than a 2 fold increase) than observed in our *in vivo* study. These results highlight the importance of obtaining tissue properties at loading rates close to the application being studied (e.g. touch vs fall) as well as reflect the potential for increased loading rate sensitivity in hydrated, live tissues compared with cadaveric tissues.

### Analysis of sex differences

We found that sex had limited effect on peak force or energy and that the results were not statistically significant. However, different trends were observed between sexes. At both frequencies the peak force and the energy absorbed were greater for males than females except for the angle 65°. At the neutral position and at 45° agrees with Teoh et al. [48] who observed no significant differences in tissue stiffness between male and female participants. However, results observed at the extreme dorsiflexion angle (65°) agrees with Abdouni et al. [47] who found that index finger tip stiffness was higher in females compared to male. The effect of sex might vary according different body locations and tissue composition at those locations. In general females have higher proportions of body fat [74, 75] than males and lower muscle mass [76–78].

### Effect of tissue thickness

Significant differences were found for peak force and energy when participants were grouped by tissue thickness but only at the extreme wrist extension and at high frequency. The lowest soft tissue thickness had the lowest peak force or energy absorbed. However, the highest soft tissue thickness did not show the highest peak force nor energy absorbed. These results are not in accordance with Garcia et al. [79] who found that plantar tissue stiffness was higher with lower plantar soft tissue thickness. However Makhsous et al. [80] who tested *in vivo* mechanical properties of ischial tuberosity, greater trochanter, posterior midthigh and biceps brachii reported that increasing the thickness of soft tissue does not necessarily lead to an increase in peak force on the tissue or even the tissue stiffness. Furthermore Ledoux et al. [72] found that energy loss varied across foot location during stress relaxation indentation testing. Additionally, they showed that the subcalcaneal location with the highest tissue thickness [81] had the least energy absorbed. This result could be explained by the fact that soft tissue responses do not always depend on soft tissue thickness. The differences of soft tissue behaviour may be due to the differences in tissue composition in different body locations. For example, tendinous tissue generally has higher stiffness [82]; therefore, soft tissue that contains more tendinous tissue tends to be stiffer. However in our case, for the same body location different tissue behaviour appeared that could be explained by the variability in tissue level properties. Indeed Choi et al. [11] concluded that the effect of age on stiffness and damping does not arise from differences in soft tissue thickness over the hip region, but instead from changes in tissue level properties. Additionally soft tissue composition and properties are affected by several others factors including: hydration [83–85], smoking [86–89], or hormone levels due to menstrual cycle phases [90–92].

We found a trend of increased energy absorption with increased tissue thickness at the neutral position and the 45° angle at both frequencies. This soft tissue behaviour was expected as during our experiment test depths were calibrated according tissue thickness. Thus greater energy absorption was expected for thicker tissues since indentation test depths were higher. However, we did not find a direct trend between tissue thickness and peak force at the other angles or frequencies, which was unexpected. Importantly we found that soft tissue properties were highly variable and that reporting and using ranges of results for soft tissue modeling is likely more valuable than using only the average results. This is consistent with previous studies using live human participants without significant preload [93].

### Compressive properties for simulations

The experimental results provided in this study provide valuable calibration and validation data for computational models of falls onto outstretched hands [94] In rigid-body dynamics simulations of falls, modelers use tissue properties to describe impacts between the body and other objects [95, 96]. The energy absorbed found in this study could help to validate the hysteresis response of the tissue which is important for quantifying the degree of elasticity or inelasticity in the impact. Demonstrating the range of variability in tissue properties in this study will also help to motivate the need to develop a range of computational models that reflect the heterogeneity of human subjects instead of drawing conclusions based on a single generic model [97–101]. The non-significant effect of position on compressive properties of the palm means that a single model can be used to represent palmar tissue for any position.

Results found at low frequency are suitable for simulations of grasping. This includes providing important input, calibration and/or validation for robotics design problems including robotic grasping, in-hand manipulation [102, 103], non-prehensile manipulation [104, 105] by controlling the contact between the gripper with other objects and providing a robust grasp strategies. In addition, computational models are being developed for ergonomic assessments of workplace activities such as grasping tools [106] and will provide important insights into product design to reduce contact stresses, discomfort, and injury risk. Although human soft tissues are broadly recognized to be viscoelastic, in finite element models of the hand, most models continue to use linear or nonlinear elastic material definitions [107]. Combining these individual tissue models into an overall biological structure such as the hand requires additional validation to ensure the assembled structure mimics real human behavior [108]. Importantly, the *in vivo* compression properties observed here may provide calibration data to derive correction factors to modify computational models to correct for post mortem derived individual tissue properties [109]. In addition, the large deformation and high rate loading data obtained in this study provide important guidance on the degree of material nonlinearity and rate dependence needed to accurately mimic *in vivo* tissues.

### Limitations

Ours is the first study to apply compression over a range of frequencies and wrist positions to determine the compressive characteristics of palmar soft tissues in living people. We used a 3 mm diameter indenter tip to characterise the soft tissue properties. However this type of tip could only measure the local properties of the soft tissue. During a forward fall the impact force would likely include more of the palmar region. Our sample size (of 15 participants) was modest, though comparable to previous studies [25, 38, 47, 110]. Additional experiments with a larger sample are warranted, and may help to establish statistically significant observations. Indentation depths were applied to approximately 50% percent of soft tissue thickness and not to an absolute depth for all participants. An absolute depth could helped us to evaluate the difference between peak force and energy absorbed according to tissue thickness. However, by applying a depth to 50% of soft tissue thickness we obtained soft tissue responses under large deformation which could not be the case by applying one absolute indentation depth on people with varied tissue thickness. Hand or thumb movement could also influence the results. Therefore, we choose to secure fingers and forearm on a custom-machined angled plates that provided stable and consistent support for the participants to keep the palm perpendicular to the indentation probe. Further work is being conducted to determine the effects of muscle activation on tissue mechanics.

## Conclusion

We found the nonlinear response of the palmar tissue had a high variability between subjects. Loading rate had the greatest effect on peak force and energy absorbed (1.7–3.0 fold increase with increased loading rate). Large deformation behaviour was significantly stiffer than small deformation behaviour. These results reinforce the need to capture the nonlinear viscoelastic characteristics of the palmar soft tissues in models of injury and emphasize the need to develop a range of computational models that reflect the heterogeneity of human tissues instead of a single generic model. Changing joint position introduced additional variability in the results but did not show statistically significant effects in a consistent manner. No significant sex differences were observed in the palmar tissue response. Significant differences were found for peak force and energy for different tissue thickness but only at extreme wrist extension and at high frequency. Overall, the results of the current study could lead to improvements in the biofidelity of computational models to simulate fall events by providing valuable data necessary to validate the compression response of palmar soft tissues during a fall onto the hands.

## Supporting information

S1 Table Representative force-displacement curves for one subject at low (A) and high (B) frequencies show marked hysteresis in the unloading responses.
(XLSX)

S2 Table A-F. Force displacement curves for subjects and angles at low and high frequency normalized to peak displacement show high inter-subject variability.
(XLSX)

S3 Table Average of stiffness at small deformation (S.D.) and large deformation (L.D.) for males, females, and all participants. (XLSX)

S4 Table A. Average of peak force (N) and energy absorbed (10^-3^J) at 31% soft tissue compression for male and female for all positions and both frequencies. B. Average of peak force at different angles for high and low frequency grouped by tissue thickness (group 1 < 2.5 mm, 2.5 mm < group 2 <3.0 mm and group 3 > 3.0 mm). C. Average of energy absorbed on different angles regarding various tissue thicknesses.
(XLSX)
